# Inhibitory effect of small interfering RNA on dengue virus replication in mosquito cells

**DOI:** 10.1186/1743-422X-7-270

**Published:** 2010-10-14

**Authors:** Xinwei Wu, Hua Hong, Jinya Yue, Yejian Wu, Xiangzhong Li, Liyun Jiang, Lei Li, Qiaoyan Li, Guoquan Gao, Xia Yang

**Affiliations:** 1 Guangzhou Center for Disease Control and Prevention, 23 Zhongshan 3rd Road, Guangzhou, Guangdong 510080, China; 2 Key Laboratory of Functional Molecules from Marine Microorganisms (Sun Yat-sen University), Department of Education of Guangdong Province, 74 Zhongshan 2nd Road, Guangzhou, Guangdong 510080, China; 3 Department of Neurology, The first hospital affiliated SunYat-sen University, 74 Zhongshan 2nd Road, Guangzhou, Guangdong 510080, China; 4 Department of Biochemistry, Zhongshan Medical School, Sun Yat-sen University, 74 Zhongshan 2nd Road, Guangzhou, Guangdong 510080, China; 5 China Key Laboratory of Tropical Disease Control (Sun Yat-sen University), Ministry of Education, Guangzhou 510080, China

## Abstract

**Background:**

Dengue viruses (DENs) are the wildest transmitted mosquito-borne pathogens throughout tropical and sub-tropical regions worldwide. Infection with DENs can cause severe flu-like illness and potentially fatal hemorrhagic fever. Although RNA interference triggered by long-length dsRNA was considered a potent antiviral pathway in the mosquito, only limited studies of the value of small interfering RNA (siRNA) have been conducted.

**Results:**

A 21 nt siRNA targeting the membrane glycoprotein precursor gene of DEN-1 was synthesized and transfected into mosquito C6/36 cells followed by challenge with DEN. The stability of the siRNA in cells was monitored by flow cytometry. The antiviral effect of siRNA was evaluated by measurement of cell survival rate using the MTT method and viral RNA was quantitated with real-time RT-PCR. The presence of cells containing siRNA at 0.25, 1, 3, 5, 7 days after transfection were 66.0%, 52.1%, 32.0%, 13.5% and 8.9%, respectively. After 7 days incubation with DEN, there was reduced cytopathic effect, increased cell survival rate (76.9 ± 4.5% *vs *23.6 ± 14.6%) and reduced viral RNA copies (Ct value 19.91 ± 0.63 *vs *14.56 ± 0.39) detected in transfected C6/36 cells.

**Conclusions:**

Our data showed that synthetic siRNA against the DEN-1 membrane glycoprotein precursor gene effectively inhibited DEN-1 viral RNA replication and increased C6/36 cell survival rate. siRNA may offer a potential new strategy for prevention and treatment of DEN infection.

## Background

Dengue viruses (DENs) are the wildest transmitted arbovirus members of the family *Flaviviridae*, genus *Flavivirus*, and compose four serotypes, DEN-1, 2, 3, and 4. As the etiologic agents, DENs can cause severe flu-like illness called dengue fever (DF), and sometimes lethal complication called dengue haemorrhagic fever (DHF) and dengue shock syndrome (DSS) [1,2]. They transmit diseases to human beings primarily through mosquitoes, mainly *Aedes aegypti *and *Aedes albopictus*. With dramatically growth in recent decades, DF affects 100 million people and results in about 25,000 deaths annually, mostly in tropical and sub-tropical regions. DHF has become a leading cause of serious illness and death among children in some Asian countries [3]. Unfortunately, effective vaccines or therapies against the infection are still not available [4].

RNA interference (RNAi) is a sequence-specific RNA degradation process in the cytoplasm of eukaryotic cells triggered by double-stranded RNA (dsRNA), widely existing in many species from nematode to human [5-8]. Upon introduction into the cells, exogenous dsRNAs are cut into 21-25 nt small interfering RNA (siRNA) by an RNase III-like enzyme called Dicer. The siRNAs form RNA-induced silencing complex (RISC) with other cellular components, and lead to the cleavage of their homologous transcript and eventually the silencing of specific gene [9-11]. RNAi is believed to be an effective endogenous mechanism for host cells to defense against virus attack [12], and has been applied as an exogenous measure to inhibit viral replication, such as HIV [13,14], influenza A virus [15], HBV [16] and SARS-CoV [17].

DEN is one of the first animal viruses that could be efficiently inhibited by RNAi [12,18]. Like other *flaviviruses*, DEN generates intracellular dsRNA as an intermediate of their replication, which may induce RNAi in the host cells. A new explanation for mosquitoes' non-pathogenic and persistent infections of DEN is that RNAi could be an important modulator [19]. Exogenous long length dsRNA corresponding to DEN sequences, introduced by either plasmid or Sindbis viruses, has been proven to mediate RNAi in mosquito C6/36 Cells and lead to inhibition of DEN replication in cultured mosquito cells [20,21]. Genetically modified *Aedes aegypti *has been raised to develop dengue virus resistance [22-24]. The mixtures of DEN specific small interfering RNAs, the hallmark of RNAi, were detected in all aforementioned studies. But little was known about the role of single siRNA with particular target sequence in the inhibition of DEN replication. Our present study was designed to investigate if a single siRNA has the inhibitory effect on DEN-1 replication in mosquito cells.

## Results

### Determination of effective siRNA sequence

**Figure 1:**
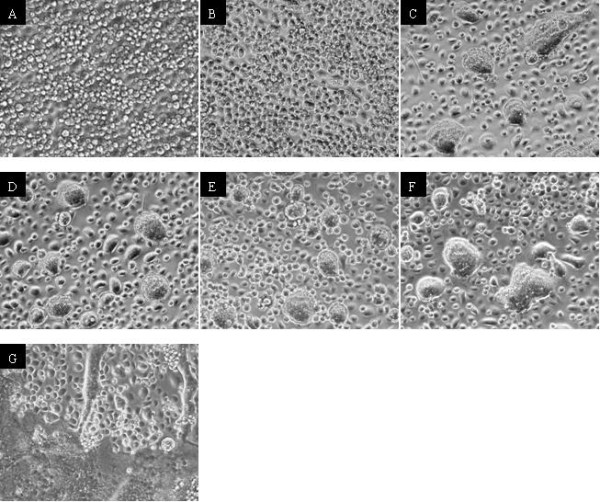
**CPE Difference in C6/36 cells transfected with four siRNAs**. Four siRNA were transfected into C6/36 cells which were challenged by DEN-1. Only DenSi-1 (B) transfected cells showed less CPE(< +) at 7 dpi than cells transfected with other siRNAs. A: normal control group; B-E: siRNA treatment group (transfected with DenSi-1-4); F: siRNA control group; G: positive control group

Four siRNA sequences (table 1) against different parts of the DEN-1 genome were designed according to the gene sequences of DEN-1 epidemic strain GZ02-218 from Guangzhou City, China 2002(GenBank access No. EF079826), and DEN-1 reference strain (GenBank access No. EU848545). Only one siRNA (DenSi-1) transfected cells showed reduced CPE(< +) after 7 days post-infection (dpi), others showed ++++ CPE, as virus positive control cells did(Figure 1). DenSi-1 was selected for further investigation.

**Table 1:** sequences and positions of designed siRNA

No	Sequence(5'-3')	Position
DenSi-1	AACGGAACCAGAUGACGUUGA	432
DenSi-2	AACUGUGCAUUGAAGCCAAAA	929
DenSi-3	AACAGGGCUAGACUUCAAUGA	1320
DenSi-4	AAGAAGAAUGGAGCGAUCAAA	133
control siRNA	UUCUCCGAACGUGUCACGUdT	--

Four siRNA sequences (table 1) against different parts of the DEN-1 genome were designed, the positions refered to DEN-1 reference strain (GenBank access No. EU848545). Negative siRNA was supplied by HiPerFect Transfection Reagent kit (Qiagen, German)

### Effects of siRNA on C6/36 survival to DEN challenge

**Figure 2:**
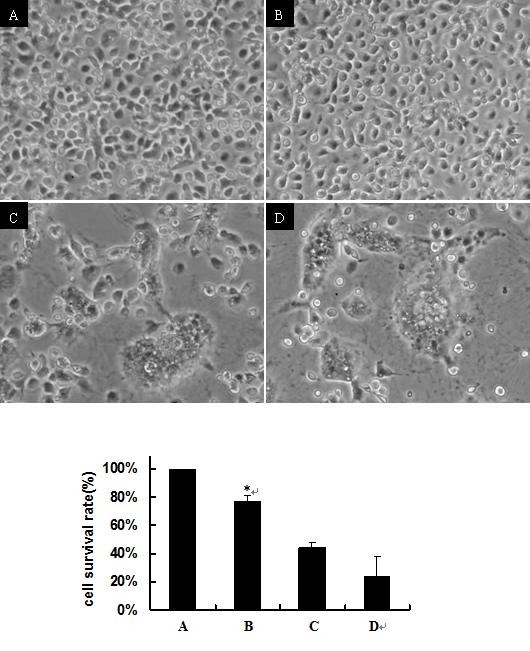
**Effects of siRNA on C6/36 cell survival rate**. C6/36 cells were transfected with DenSi-1(B) or control siRNA(C), and challenged by DEN-1(B-D). CPEs were observed and cell survival rates were measured by MTT assay at the 7^th ^dpi. Compared with virus positive control group, the cell survival rate of siRNA group increased by 2.26-fold (n = 5, P < 0.05), but siRNA control group showed no significant difference (n = 5, P > 0.05) A: normal control group; B: siRNA treatment group; C: siRNA control group; D: virus positive control group; Up: CPEs of C6/36 cells at the 7th dpi; Down: MTT assay results for C6/36 cells survival rate at the 7th dpi (n = 5). * P < 0.05, compared with virus positive control group.

CPE in each group was observed at the 7^th ^dpi. The virus positive control group and control siRNA group showed a large number of CPE up to ++++, characterized by cell swelling and fusing, and reduced cell number; while normal group and siRNA group showed no CPE and no observable decrease in cell number. The cell survival rate of C6/36 cells, measured by MTT assay, of siRNA group was 76.9% ± 4.5%, control-siRNA group was 43.9% ± 3.6%, and virus positive control group was 23.6% ± 14.6%. Compared with virus positive control group, the cell survival rate of siRNA group increased by 2.26-fold (n = 5, P < 0.05), but siRNA control group showed no significant difference (n = 5, P > 0.05) (Figure 2).

### Effects of siRNA on viral loading in infected C6/36 cells

The amount of intracellular DEN-1 viral loading was detected by Real time RT-PCR at the 7^th ^dpi. The CT value of DEN-1 RNA of siRNA group was 19.91 ± 0.63, control-siRNA group was 14.63 ± 0.91, virus-positive control group was 14.56 ± 0.39, and no viral RNA detectable in normal control group. Compared with the virus positive control group, siRNA group showed significant decrease of viral loading (n = 5, P < 0.05), but control siRNA group showed no significant difference (n = 5, P > 0.05) (table 2). The viral RNA amount of siRNA group was reduced for 2^5.34 ^fold (about 40 fold), the inhibition rate was 97.54%.

**Table 2:** DEN-1 viral RNA load in C6/36 cells at the 7^th ^dpi (n = 5)

	Normal control group	siRNA group	Control siRNA group	Positive control group
CT value	Negative	19.91 ± 0.63*	14.63 ± 0.91	14.56 ± 0.39
ΔCT value	--	5.34	0.07	--

* P < 0.05, Compared with the virus positive control group

The amount of intracellular DEN-1 viral loading was measured by Real time RT-PCR at the 7^th ^dpi.

### siRNA transfection efficiency and stability

**Figure 3:**
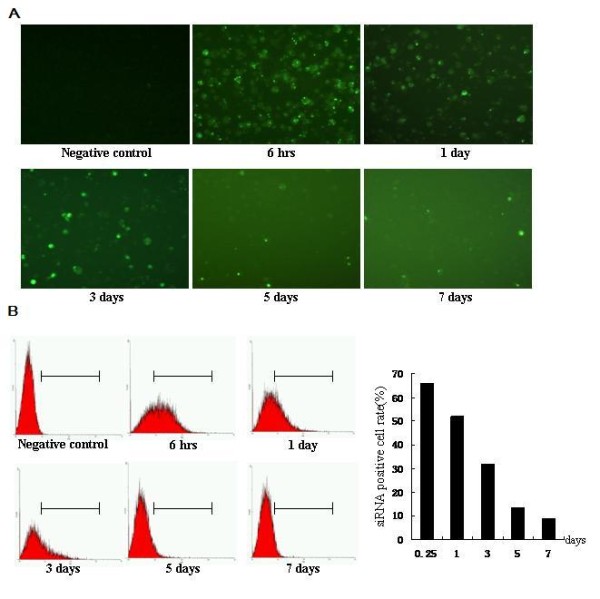
**Stability of transfected siRNA in C6/36 cells**. A: C6/C36 cells were transfected with 1.0 μg FAM-labeled DenSi-1 and cultured for 7 days. FAM fluorescence in the cells was observed under fluorescence microscope(×200); B: C6/36 cells transfected with FAM-labeled siRNAs were harvested at different time points as indicated, washed and resuspended with PBS (pH7.4). FAM fluorescence was quantified by flow cytometry and the percentage of fluorescence positive cells was measured. The rates of C6/36 cells containing FAM-labeled siRNA at 6 hours, 1, 3, 5, and 7 dpi were 66.0%, 52.1%, 32.0%, 13.5% and 8.9%, respectively.

After transfection with FAM-labeled siRNA, fluorescent signals can be detected in more than half of C6/36 cells cytoplasm under a fluorescent microscope. During the incubation, the fluorescent intensity in the cells gradually decreased (Figure 1). And the percentage of FAM positive cell at 6 h, days 1, 3, 5, and 7 were 66.0%, 52.1%, 32.0%, 13.5%, and 8.9%, respectively, by flow cytometry (Figure 3).

## Discussion

As a rapid developing technology, RNAi has not only become a powerful tool for studying gene function and development of gene-based therapies, but also been widely used in anti-virus researches. Precious studies suggested that, at the molecular, cellular and individual levels, RNAi can potentially be used to block viral transmission and thus prevent the viral diseases [12,18]. With the high efficiency, specificity and low cytotoxicity, RNAi offered a new promise of anti-viral therapy.

For ssRNA viruses such as DEN, there genomes are exposed within cytoplasm and become potential targets for RNAi. This likely happened at the moment between the uncoating of viral RNA and the viral replication [25]. Virus resistance has been proven to be generated by RNAi triggered from exogenous DEN-specific dsRNA. Adelman *et al. *[20] showed this resistance by a 290 nt dsRNA homologous to the type II dengue virus PrM gene which was expressed by plasmid, in cultured mosquito C6/36 cells. Travanty *et al. *[23] developed transgenic mosquito lines that transcribed the same 290 nt dsRNA with insect promoters, but failed to express in critical site for DEN replication such as midguts. Franz *et al. *[24] increased the size of dsRNA to 578 nt and succeeded in midgut expression and viral transmission diminishing. In addition to DEN-2, alternation of the replication kinetics of DEN-1 has also proven to be triggered by dsRNA in C6/36 cells [26].

However, long dsRNA fragments are associated with higher cost in synthesis, poorer stability, and raise the chance for mismatch rate. At the same time, introduction of dsRNAs longer than 30 base pairs into mammalian cells will activate the potent interferon and protein kinase R antiviral pathways, resulting in non-sequence-specific effects that can include apoptosis [27]. Therefore, these disadvantages largely restrict the use of long dsRNA in pre-clinical and clinical applications.

siRNAs are degraded products of dsRNA with 21-25 nt in length, and have no such disadvantages because of their short length. They are also described as "hallmark" of RNAi in all previously published papers. We hypotheses that the siRNA derived from the same fraction can have the same inhibitory effect as the dsRNA did. Therefore, in this study, we designed and synthesized 21 nt siRNAs against the DEN-I viral PrM gene, and investigated their inhibition effects of dengue virus replication in transfected C6/36 cells. In four siRNAs we designed, only one showed the function to reduce CPE after DEN infection. As expected, the location of this siRNA in DEN-1 is inside the corresponding location of 290 nt dsRNA reported before in DEN-2.

With transfection of the selected siRNA, C6/36 cells showed reduced CPE, and increased cell survival by 2.26 folds and eliminated viral RNA by about 97.54% compared to virus infection only group, at the 7^th ^dpi. These data indicate that siRNA against DEN viral genome can effectively inhibit viral RNA replication in the C6/36 cells, protect host cell from viral attack, suggesting its potential role in prevention and treatment of dengue fever.

Recent study have shown [19] that *Aedes aegypti *can produce dsRNAs homologous to dengue viral genes and trigger an intrinsic siRNA anti-viral action, but this endogenous anti-viral mechanism can not effectively inhibit the replication of dengue virus. Data presented in our study revealed that exogenous siRNA in *Aedes albopictus *cells is effective in inhibiting viral DNA replication. Therefore, it is possible that the anti-viral mechanisms mediated by exogenous and endogenous siRNAs may act synergistically in the protection of cells from viral attacks, although this hypothesis needs further researches to prove.

Because the short siRNAs are unstable inside the cells, and also diluted by continuous cell division, the siRNA contents in the cells decline over time resulting in the weakening of interference effect. From the data in this study, siRNAs can be successfully transfected into 66% of the cells but this percentage gradually reduced with time and siRNA molecules only retained in 8.9% of the cells at the seventh day post transfection. Therefore, maintaining the continuity of RNA interference is crucial for the promotion and application of RNAi technology. Alternative form of vectors, such as retrovirus[28] and nano device[29], will be applied in the future.

Since currently there are no effective therapies or vaccines against Dengue fever, the use of RNAi to suppress dengue virus replication and protect cells from viral attacks will undutiful provide a new research strategy for the prevention and treatment of this disease.

## Conclusions

Our data showed that synthetic siRNA against the DEN membrane glycoprotein precursor gene effectively inhibited DEN viral RNA replication and increased C6/36 cell survival rate. siRNA may offer a potential new strategy for prevention and treatment of DEN infection. The stability and inhibitory efficiency of siRNA need further improvement in the future.

## Methods

### 1. Cell culture and virus replication

Type 1 DEN strain GZ02-218 and *Aedes albopictus *C6/36 cell line were both from the Department of Virology&Immunology in Guangzhou Center for Disease Control and Prevention. C6/36 cells were grown at 28°C, in Eagle's minimal essential medium (MEM, Gibco, USA) supplemented with 10% fetal bovine serum (FBS, Gibco, USA), 100 μg/mL penicillin and streptomycin, pH 7.4(maintain medium). Cells were passaged every 5 to 7 days to maintain exponential growth. DEN-1 strain GZ02-218 was passaged by infecting monolayers of C6/36 cells and viruses were harvested at 12-14 days. TCID_50 _of virus was measured for viral challenge.

### 2. siRNA design and synthesis

Four pairs of siRNAs against different parts DEN-1 viral genome were designed online (Qiagen, German) based on the common sequences of the epidemic strain GZ02-218 in Guangzhou City of China in 2002 (GenBank access number EF079826), DEN-1 reference virus strain (GenBank access number EU848545), and other DEN strains. Four siRNA fragments (DenSi-1~4) were synthesized and purified by PAGE electrophoresis (Ambion, USA). siRNAs was also labeled with FAM and purified for visualization after transfection (Ambion, USA). A siRNA fragment inconsistent containing sequence with DEN was used as the negative control (Qiagen, German).

### 3. Experimental groups

C6/36 cells were cultured in 24-well plate and divided into the following four groups based on different treatments: normal control group(A): cells received no siRNA transfection or viral infection; siRNA treatment group(B): cells transfected with siRNA and subsequently challenged by DEN-1 (GZ02-218); siRNA control group(C): cells transfected with the control siRNA having no common sequence with dengue virus genome and challenged by DEN-1; and virus positive control group(D): cells received no siRNA transfection but directly challenged by DEN-1. Every group contained 6 wells of cells.

### 4. siRNA transfection and viral infection

C6/36 cells were cultured in maintain medium at 28°C, and then inoculated in 24 well plates at 1 × 10^6 ^/well in 0.5 ml medium the day before transfection. When 80% ~ 90% cells grew into monolayer, they were transfected with siRNA molecules according to the manual for HiPerFect Transfection Reagent kit (Qiagen, German). 1.0 μg siRNA and 3 μl HiPerFect Transfection Reagent were used for each well. After cultured at 28°C for 4 hours, the medium with transfection reagent was removed. Cells were washed by MEM, challenged by 100 TCID_50 _DEN-1 and cultured at 33°C in maintain medium for further investigation.

### 5. CPE effects of Dengue virus on C6/36 cells

The cytopathic effect (CPE) of DEN-1 infection on C6/36 cells, including cell rounding, syncytium formation and cell death, was evaluated under a light microscope, and scored based on the severity from 0 (no CPE observed, no cell death) to the most severe level ++++ (CPE observed in 100% cells).

### 6. MTT assay for cell survival rate

At the 7^th ^dpi, MTT assay was applied to measure cell viability. 100 μl MTT was added to every well and incubated for 4 hours then replaced with 1 ml DMSO. The 24-well plate was shaken at 37°C for 10 minutes and OD_490 _of every well was obtained in a microplate reader. The OD value obtained from the normal control group was set as 100% viability, and the cell survival rate of other groups was calculated as their respective OD value divided by that of the control group.

### 7. Real-time RT-PCR detection of dengue virus RNA in C6/36 cells

At the 7^th ^dpi, C6/36 cells were collected and viral RNA was isolated with QIAamp Viral RNA Extraction Kit (Qiagen, German), and quantified with the dengue virus real-time fluorescent RT-PCR detection kit (Shenzhen Taitai Genomics, China). PCR reaction conditions were: one cycle of 50°C for 30 min and 95°C for 3 min, then 40 cycles of 95°C for 5 sec and 60°C for 40 sec. The relative amount of viral load in each group was represented by CT value, and the changes between groups were calculated by comparative CT (ΔCT) values.

### 8. Transfection efficiency and stability of siRNA

C6/36 cells were transfected with FAM-labeled siRNAs and cultured for 7 days. Fluorescence signal was observed under fluorescence microscope at 6 hours, 1, 3, 5, and 7 days. Cells were harvested at every time point, and measured by flow cytometry. siRNA positive cell rate was calculated as the percentage of cells containing fluorescent signals.

### 9. Statistical analysis

All values were presented as mean ± S.D. Statistical significance was evaluated using the two-tailed Mann-Whitney U-test; P < 0.05 was considered significant.

## Competing interests

The authors declare that they have no competing interests.

## Authors' contributions

WX and HH performed majority of the experiments and wrote the part of material and methods. YJ, WY and LX performed siRNA transfection experiments. JL, LL and LQ cooperated on cell and virus cultures. YX and GG designed the experiments and wrote the manuscript. All authors read and approved the final manuscript.
